# Hexosamine Biosynthetic Pathway and Glycosylation Regulate Cell Migration in Melanoma Cells

**DOI:** 10.3389/fonc.2019.00116

**Published:** 2019-03-05

**Authors:** Rafaela Muniz de Queiroz, Isadora Araújo Oliveira, Bruno Piva, Felipe Bouchuid Catão, Bruno da Costa Rodrigues, Adriana da Costa Pascoal, Bruno Lourenço Diaz, Adriane Regina Todeschini, Michelle Botelho Caarls, Wagner Barbosa Dias

**Affiliations:** ^1^Laboratório de Glicobiologia Estrutural e Funcional, Centro de Ciências da Saúde, Instituto de Biofísica Carlos Chagas Filho, Universidade Federal do Rio de Janeiro, Cidade Universitária, Rio de Janeiro, Brazil; ^2^Laboratório de Inflamação, Centro de Ciências da Saúde, Instituto de Biofísica Carlos Chagas Filho, Universidade Federal do Rio de Janeiro, Cidade Universitária, Rio de Janeiro, Brazil; ^3^Laboratório de Matriz Extracelular, Centro de Ciências da Saúde, Instituto de Ciências Biomédicas, Universidade Federal do Rio de Janeiro, Cidade Universitária, Rio de Janeiro, Brazil

**Keywords:** hexosamine biosynthetic pathway, *O*-GlcNAcylation, glycosylation, cell migration, melanoma, cancer

## Abstract

The Hexosamine Biosynthetic Pathway (HBP) is a branch of glycolysis responsible for the production of a key substrate for protein glycosylation, UDP-GlcNAc. Cancer cells present altered glucose metabolism and aberrant glycosylation, pointing to alterations on HBP. Recently it was demonstrated that HBP influences many aspects of tumor biology, including the development of metastasis. In this work we characterize HBP in melanoma cells and analyze its importance to cellular processes related to the metastatic phenotype. We demonstrate that an increase in HBP flux, as well as increased *O*-GlcNAcylation, leads to decreased cell motility and migration in melanoma cells. In addition, inhibition of *N*- and *O*-glycosylation glycosylation reduces cell migration. High HBP flux and inhibition of *N*-glycosylation decrease the activity of metalloproteases 2 and 9. Our data demonstrates that modulation of HBP and different types of glycosylation impact cell migration.

## Introduction

Metastatic melanoma is a devastating disease, and the most dangerous type of skin cancer. Melanoma's incidence increases and is associated with the main risk factor for the disease, UV exposure (1). Tumors diagnosed in early stages of the disease are surgically removed, the lethality of the disease is mostly associated with late diagnosis and metastatic disease in patients (2). The main goal of the last decade of melanoma research was to identify genes/proteins that could be used as possible targets for therapy. Although the recent introduction of targeted therapy and immune checkpoint inhibitors revolutionized the prognosis of some patients, the available treatments are still not ideal due to the emergence of drug resistance and/or low response rates (3). To understand the mechanisms responsible for cell migration and therefore metastasis are of foremost importance to further develop effective drugs to treat, or even prevent the metastatic stage.

Cancer cells present altered metabolism, especially the pathways involved in energy production like glycolysis, a phenomena known as the Warburg effect (4). Metastatic melanoma presents with an increase in both glycolysis and oxidative phosphorylation, giving the melanoma a metabolic advantage to thrive in different environments (5). We reason that not just glycolytic flux, but also the pathways linked to it, such as the pentose phosphate pathway, lactic acid fermentation and hexosamine biosynthetic pathway (HBP) will be altered in tumor cells. The HBP is a branch of glycolysis responsible for the production of UDP-*N*-acetylglucosamine (UDP-GlcNAc). Its final product is considered an important nutrient sensor, since HBP uses as substrates molecules from carbohydrate (glucose), lipid (acetyl-CoA), amino acid (glutamine), and nucleotide (UTP) metabolism to generate UDP-GlcNAc (6). This metabolite is an activated monosaccharide used as substrate by glycosyltrasferases in glycosylation reactions of proteins and lipids, or to generate UDP-GlcNAc-derived activated monosaccharides, also used for glycosylation, such as UDP-GalNAc and CMP-Neu5Ac (7). It is well-established that aberrant glycosylation is a hallmark of cancer (8, 9) and because glycosylation depends on the production of UDP-GlcNAc, it is reasonable to assume that this pathway is important to tumorigenesis and tumor progression.

Glycosylation of extracellular proteins, by *N*- and *O*-glycans, form what is called the glycocalix, a saccharide coating in cells formed by glycoproteins and glycolipids that mediates cell-cell and molecules-cell interactions. Secreted proteins are also glycosylated and contribute to the composition of the extracellular matrix (10). In mammalian cells, oncogenic transformation is commonly accompanied by changes in the glycosylation profile of proteins and lipids and is directly involved in the increase of metastatic potential and the spread of tumors (11). In the case of cancer, there are changes in O-glycosylation (12), in N-glycosylation (13), and glycolipids (14). Altered O-glycosylation is a universal feature of cancer cells. Truncated O-glycosylation (T and Tn antigen) are the tumor-associated carbohydrate antigens most found and its expression highly correlate with cancer progression and metastasis (15, 16). Changes in the oligosaccharide structure of N-glycans have also been described in several tumors (15). Augmented expression of N-acetylglucosaminyltransferase V (Mgat5) results in branched *N*-glycans with higher affinity for galectins playing a role in cell growth and metastases (15). The GlcNAc adjacent to Asn in the core can be modified by the action of α1-6-fucosyltransferase (FUT8). The upregulation of core fucosylation (FUT8) and downregulation of a-1,2fucosylation were recently identified as features of metastatic melanoma. Agrawal et al. showed that FUT8 facilitates invasion and tumor dissemination (17). *N*- and *O*-Glycans can be further decorated by the addition of Gal, GalNAc, GlcNAc Fuc, and sialic acid. Recently, Sweeney and colleagues demonstrated that melanomas downregulate the glycosyltransferase beta-1,6-N-acetylglucosaminyltransferase (GCNT2) promoting melanoma xenograft growth, colony formation, and cell survival (18). In addition, glycans from tumor cells present increased sialic acid associated with cancer progression, occurrence of metastasis, poor prognosis and therapeutic (15). Due to its expression in tumor cells, its absence in normal tissues, combined with the function of glycoconjugates in cellular biology these tumor-associated glycoproteins are generally biomarkers targets for immunotherapy and development of vaccines for cancer (16).

Glycosylation of intracellular proteins, called *O*-GlcNAcylation, is characterized by addition of an *N*-acetylglucosamine (GlcNAc) from the precursor UDP-GlcNAc to serine and threonine residues on proteins. The enzyme *O*-linked β-*N*-acetylglucosamine transferase (OGT) catalyzes the addition of GlcNAc to proteins, whereas the enzyme *O-*GlcNAcase (OGA) removes the sugar. Unlike extracellular glycosylation, *O-*GlcNAc is not elongated into more complex structures and is localized mainly in nucleocytoplasmic compartments (19). O-GlcNAcylation regulates many different aspects of protein function, such as enzymatic activity, protein stability and subcellular localization, and is thus intrinsically related to cell signaling and metabolism (19). It is well-established that *O-*GlcNAc levels and protein expression of OGT and OGA are aberrant in different tumors and may be associated with prognosis and tumor grade (9).

Changes in the HBP flux, which can be achieved through changes in nutrient availability (20, 21) or presence of cytokines (22), affect protein *O*-GlcNAcylation (23) as well as *N*- and *O*-glycosylation (24). The extension of HBP's influence in tumor cell behavior is not well-understood, but a few papers have investigated the correlation between this pathway and tumor cell biology through analyzing its rate-limiting enzyme, Glutamine: Fructose-6-phosphate amidotransferase (GFAT). GFAT1 and GFAT2 are over expressed and its expression is associated with a severe prognosis in different tumor types (25–29), while its decrease is associated with a good prognosis in other tumors (30–32). These data suggest that tumor cells possess altered flux through HBP and that the regulation of this pathway in tumorigenesis differs between cell types. Additionally, two recent studies demonstrate the importance of HBP to tumor cells and especially to metastasis (26, 33). Thus, the aim of this work is to investigate the influence of HBP and of glycosylation (*O*-GlcNAcylation, *N*-glycosylation and *O-*GalNAc glycans) in two human melanoma cell lines.

Here, we describe the HBP status in melanoma cells as well as their glycosylation profile and show that increase in HBP's flux as well as hyper-*O*-GlcNAcylation decreases cell motility and migration of melanoma cells. In addition, we demonstrate that inhibition of *N-*glycans and *O-*GalNAc glycans leads to the same phenotype. These data show the importance of those pathways for cell movement, especially in the case of more aggressive cells, where cell migration has an important function. Lastly, we observe that HBP and *N*-glycosylation also impacts matrix metallo proteases' (MMPs) activity as an independent mechanism in the modulation of cell migration.

## Experimental Procedures

### Chemicals and Reagents

All primary and secondary antibodies used for immunoblotting were used at 1:1,000 and 1:2,000 dilutions, respectively. Anti-*O-*linked *N*-acetylglucosamine antibody (RL2) (#sc-59624), anti-OGT (#sc-32921), anti-GFAT1 (#sc-134894), and anti-GFAT2 (#sc-134710) were purchased from Santa Cruz Biotechnology. Anti-β-tubulin (#86298T), anti-GFAT (#5322S), anti-rabbit-HRP (#7074S) were purchased from Cell Signaling. Anti-mouse HRP (#NA931V) was purchased from GE life sciences. Thiamet-G (TMG) (#110165CBC) was purchased from CalBiochem. Anti-OGA (SAB4200311), streptavidin-Cy3 (#S6402), gold (III) chloride trihydrate (#520918), tunicamycin (TM) (#T7761) and benzyl2-acetamido-2-deoxy-α -D-galactopyranoside (BAG) (#B4894) and glucosamine (#G1514) were purchased from Sigma-Aldrich.Lectins (AAL, #B-1395; E-PHA, #B-1125; L-PHA, #B-1115; MAA, #B-1265; PNA, #B-1075; SNA, #B-1305; VVL, #FL-1231; WGA, #B-1025) and anti-Sialyl Lewis an antibody (#VP-S280)were purchased from Vector.

### Cell Culture

Human melanoma cancer cell lines WM983A and WM852 (provided by Dr. Meenhard Herlyn, The Wistar Institute, Philadelphia, PA) were grown in 3:1 Dulbecco's modified Eagle's Medium/Nutrient Mixture F12 ham (DMEM-F12, Sigma) and L-15 Medium (Sigma) supplemented with 10% heat-inactivated fetal bovine serum (FBS), 100 units of penicillin, and 100 mg/ml streptomycin (#P433, Sigma), at 37°C with 5% CO_2_. To induce human GFAT2 over expression, WM852 cells were transfected with 100 ng of plasmid for GFAT over expression using the transfection reagent FuGENE HD (Promega #E2311).

### Immunoblotting

Cells were washed with phosphate-buffered saline and homogenized in lysis buffer (150 mm NaCl, 30 mm, Tris-HCl, pH 7.6, 1 mm EDTA, 1 mm EGTA, 0.1% SDS, 1 mm phenylmethylsulfonyl fluoride, and 200 mMGlcNAc) with the addition of protease inhibitors. Samples were centrifuged and the supernatant was collected, protein concentration was determined and modified Laemmli buffer was added. Samples were separated on SDS-polyacrylamide gels and were subsequently transferred to nitrocellulose membrane (Bio-Rad). The membranes were blocked in Tris-buffered saline with 0.1% (v/v) Tween 20 with 3% (w/v) bovine serum albumin. The blocked membranes were then incubated overnight at 4°C with primary antibodies. The blots were then washed, incubated with the appropriate secondary antibody, developed using ECL (GE Healthcare), and exposed to Image Quant LAS 4000 (GE Healthcare). ImageJ software was used for densitometry analysis of immunoblots and measurements were normalized against β-tubulin as loading control.

### GFAT Enzymatic Activity

PBS washed-pellets corresponding to 1 × 10^6^ cells were homogenized in buffer A [PBS pH 7.4 containing 50 mM KCl, 5 mM EDTA, 1 mM dithiothreitol (DTT), 1 mM PMSF, and protease inhibitors] followed by sonication. Cell extracts were centrifuged at 12,000 × g for 10 min at 4°C, and the protein in supernatant were quantified using Bradford method (34). Cell lysates containing 50 μg of protein in each condition were incubated in PBS pH 7.4, 1 mM DTT and 5 mM EDTA, and with GFAT substrates [10 mM fructose-6-phosphate, 10 mM L-glutamine (Gln)], for 1 h at 37°C under agitation. The preexistent GlcN6P in cell extract were discounted by incubating 50 μg of protein in the same reaction buffer but without GFAT substrates.

The formation of glucosamine-6-phosphate (GlcN-6P) was determined by the assay described by Elson & Morgan (35), with the modifications described by Qian et al. (36). Briefly, 10 μL of 1.5% acetic anhydride (Sigma, USA) and 50 μL of 100 mM sodium tetraborate was added to 100 μL of reaction mixture and incubated at room temperature for 5 min under agitation. The samples were then incubated at 80°C for 25 min, cooled down at 4°C for 5 min, and mixed with 130 μL of Ehrlich reagent (10% *p*-dimethylaminobenzaldehyde, 11% HCl, 1.5% water in acetic acid, diluted 1:2 in acetic acid immediately before use) in a 96 wells microplate. The color was developed for 30 min at 37°C and finally read at 585 nm in microplate reader (SpectraMax 190, Molecular Probes, USA). The absorbance of the samples not incubated with GFAT substrates was discounted and the concentration of GlcN-6P was determined comparing the resultant absorbance of the samples with GlcN-6P standards processed in the same manner.

### UDP-GlcNAc Quantification

The cell extracts were processed according previous work (22). Briefly, polar metabolites were extracted from 1 × 10^6^ cells with chloroform, methanol and water (2:2:1.8). Polar fractions were dried by centrifugation under vacuum (Speed Vac) and solubilized in water to a final concentration of 10 μg/μL. All the samples were spiked with 0.2 mM p-nitrophenol (pNP) as external standard. Twenty microliters of each sample was subjected to chromatographic separation utilizing a Hypercarb PGC column (2.1 × 100 mm, Thermo Fisher Scientific, USA) running in HPLC Shimadzu ETC. Sugar nucleotides were eluted using discontinuous linear gradient of mobile phases A (0.2% formic acid and 0.75% ammonium hydroxide in water) and B (95% acetonitrile with 0.1% formic acid and 0.07% ammonium hydroxide) at a flow rate of 0.3 mL/min. UDP-GlcNAc quantification was made using a calibration curve.

### Glycan's Profile

The changes in cell surface glycoconjugates were analyzed by flow cytometry using specific lectins and antibody (Table 1). Cells were washed three times with PBS and fixed in a 3.7% formaldehyde solution in PBS. The cell surface was blocked in a PBS-BSA3% solution and incubated with biotinylated lectins (20 μg/ml) or anti-Sialyl Lewis an antibody (1:100) overnight. Samples were then washed three times with PBS and incubated for 1 h with avidin-Cy3 (1: 2,500) or secondary antibody-FITC (1:2,000) then washed two times with PBS. Probed samples were further analyzed by flow cytometry (BD Biosciences FACS Calibur) and the data were analyzed using Flow Jo software (Tree Star).

**Table 1:** Panel of lectins and antibody used for the characterization of glycan's profile.

**Name**	**Specificity**	**Fluorochrome**
AleuriaAurantialectin (AAL)	Fucose α-linked	Cy3 (FL-2)
Maakiaamurensis (MAA)	Neu5Ac α2,3-linked	Cy3 (FL-2)
Peanut agglutinin (PNA)—T antigen	Gal β1,3-GalNAc	Cy3 (FL-2)
Phaseolus Vulgaris erythroagglutinin (E-PHA)	Gal β1,4-GlcNAc β1,2-Man	Cy3 (FL-2)
Phaseolus Vulgaris leukoagglutinin (L-PHA)	GlcNAc β1,6-Man	Cy3 (FL-2)
Sambucusnigraagglutinin (SNA)	Neu5Ac α2,6-linked	Cy3 (FL-2)
Anti-Sialyl Lewis antibody	Sialyl Lewis a	FITC (FL-1)
Vicia Villosalectin (VVL)—Tnantigen	α-GalNAcpeptide	FITC (FL-1)

### Wound-Healing Assay

Cells were plated (1 × 10^5^ cells/well) in 24-well-plate in duplicates for each condition. On the next day a scratch was made in the confluent well using a plastic pipette tip, after which the well was washed with PBS 3 times to remove cell debris. New media containing 2% FBS, with or without the addition of GlcN, TMG, TM or BAG was added to each well. Micrographs of the wells were taken at the beginning (0 h) and at the end of the experiment (20 h). ImageJ software was used for quantification of cell migration into the wound. Migration was measured as percentage of the wound area in control (0 h), which was considered 0% of migration.

### Colloidal Gold Phagokinetic Track Assay

Single cell motility was measured by this colloidal gold phagokinetic track assay (37). The colloidal gold-coated plate was prepared using 24-well-plate. Briefly, wells were incubated with PBS ethanol 30%-BSA1% overnight followed by the addition of colloidal gold solution (gold chloride trihydrate 14.5 mM, sodium carbonate 26,5 mM and 0.1% formaldehyde). On the next day the wells were washed with culture media and cells were plated (7 × 10^3^ cells/well), micrographs were taken 18 h after seeding. ImageJ software was used for quantification of cell tracks (37).Only single cell and clear tracks were considered for quantification.

### Gelatin Zymography for MMP-2 and MMP-9 Activity

Cells were seeded (5 × 10^5^ cells/well) in 6-well-plate. On the next day, the cell monolayer was washed with sterile PBS to remove serum completely and cells were incubated with serum-free media at 37°C for 24 h. The media was collected and centrifuged (400 × g, 5 min at 4°C) to remove cells and debris. 75 μL of the clarified supernatant was mixed with 25 μL of sample buffer and loaded 10 μL per lane in a 7.5% polyacrylamide gel containing gelatin. The gel was washed 2 x 30 min with washing buffer (2.5% Triton X-100, 50 mM Tris-HCl pH 7.5, 5 mM CaCl_2_; 1 μM ZnCl_2_) and rinsed for 10 min in incubation buffer at 37°C with agitation (1% Triton X-100, 50 mM Tris-HCl pH 7.5, 5 mM CaCl_2_; 1 μM ZnCl_2_). After that, the fresh incubation buffer was replaced and incubated for 24 h at 37°C. The gel was stained with staining solution for 1 h (40% Methanol, 10% acetic acid, 0.5% Coomassie blue). Next, the gel was rinsed with H_2_O and incubated with destaining solution (40% methanol, 10% acetic acid) until bands can clearly be seen. The gel bands were photographed and analyzed by ImageJ software.

### Statistical Analysis

All the data reported in this paper were expressed as the mean ± S.D. from at least three independent experiments. A significant difference from the respective control for each experimental test condition was assessed by one-way analysis of variance or Student's *t*-test using GraphPad Prism 6.0 software. Values of *p* < 0.05 were considered statistically significant.

## Results

### Analysis of HBP Flux in Melanoma Cells

**Figure 1:**
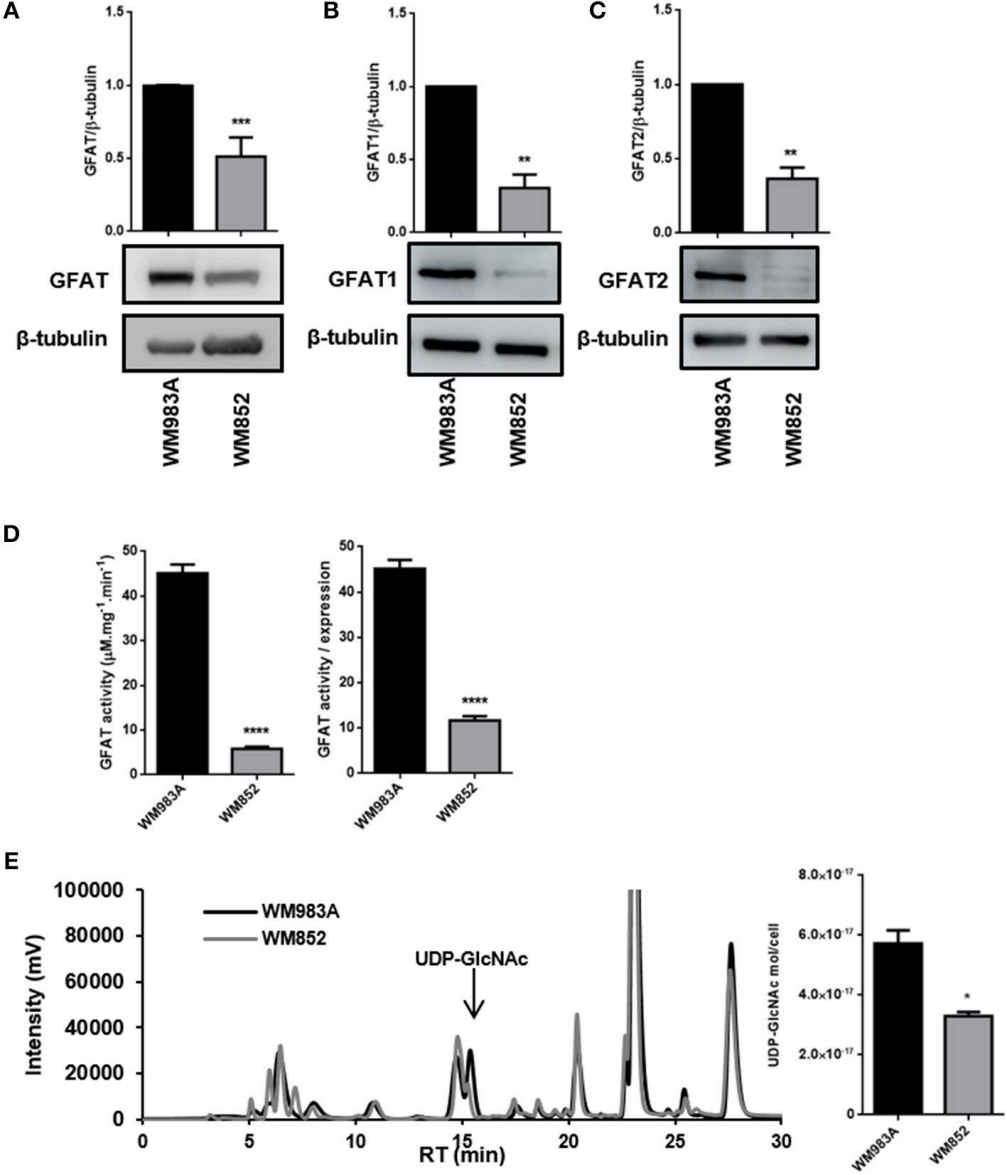
HBP's status in melanoma cell lines. **(A)** Protein levels of total GFAT, **(B)** GFAT1, and **(C)** GFAT2 were measured by western blotting in WM983A (black bars) and WM852 (gray bars) cell lines. Quantification of protein levels in each cell line was normalized to β-tubulin. **(D)** Total and relative GFAT activity was measured in cell lysates by a colorimetric assay. Global GFAT activity was normalized by global GFAT expression in order to isolate activity from expression levels. **(E)** Quantification of UDP-GlcNAc by cell number and representative chromatogram showing UDP-GlcNAc peak in the two cell lines. All experiments were performed with at least 3 biological replicates. *p < 0.05; **p < 0.01; ***p < 0.001; ****p < 0.0001.

In order to analyze HBP's flux in melanoma cells we used two human melanoma cell lines WM983A and WM852. WM983A is derived from a primary tumor, while WM852 cells are derived from an aggressive tumor (abdominal metastasis). The HBP status was evaluated by the expression of GFAT, the rate-limiting enzyme of the pathway, and by the production of UDP-GlcNAc, the final product of HBP. GFAT has two main isoforms: GFAT1, which is ubiquitously expressed among different organs, and GFAT2, found in normal conditions mostly in the heart, nervous and reproductive system, but found as well in tumor cells outside the brain (26, 38). The pattern of expression of GFAT1 and GFAT2 is not well-known in normal or tumor skin cells. We observe that both GFATs are different expressed in melanoma tumor cells (Figures 1A–C). When comparing cells lines we observe that in WM852 cells protein levels of total GFAT (including isoforms 1 and 2) are decreased by 50% (Figure 1A). The same pattern is observed when using an antibody specific for GFAT1 (Figure 1B) and GFAT2 (Figure 1C). Not only the expression of GFAT1 and 2 is decreased, but also total activity of the enzyme is significantly decreased in WM852 cells, as measured by the formation of GlcN-6P (Figure 1D).

Finally, to confirm that HBP's flux decreased in WM852 cells, as suggested by the decreased expression and activity of GFAT, we quantified the amount of UDP-GlcNAc in each cell line. Indeed, the amount of UDP-GlcNAc in WM852 is significantly lower than the pool found in WM983A (Figure 1E).

### Glycan Profile Characterization in WM983A and WM852 Melanoma Cells

**Figure 2:**
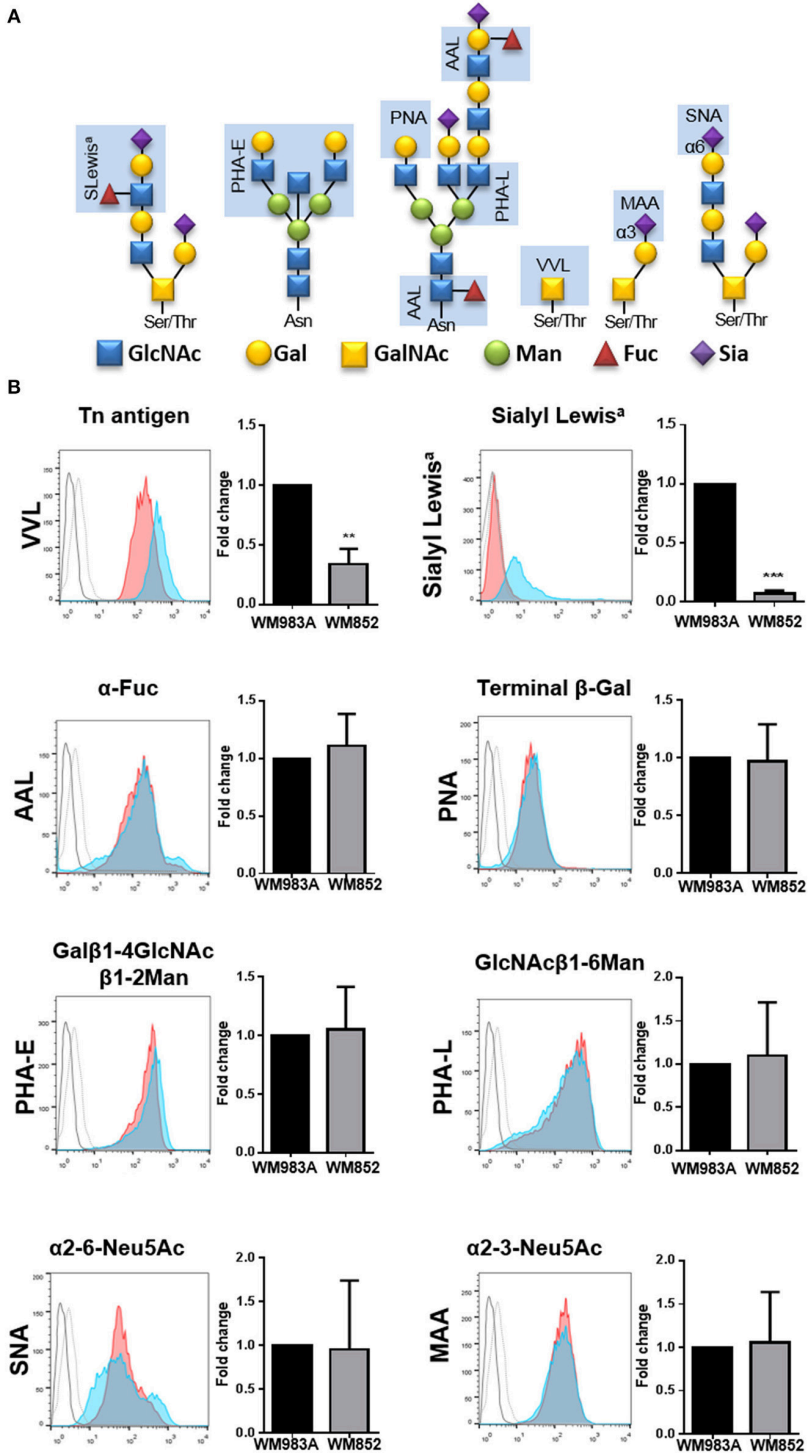
Glycan's profile of melanoma cell lines. **(A)** Scheme representing binding specificities of lectins and antibody used in the experiment (light blue rectangle). **(B)** Surface glycans of WM983A and WM852 melanoma cells were analyzed and quantified. Bar graph and representative histograms comparing the fold change in fluorescence intensity for each glycan epitope in WM983A (red) and WM852 (blue). Dotted line (WM983A) and full line (WM852) refers to cells stained without the lectin or primary antibody. MIF values found in WM852 cells were normalized by the expression found in WM983A cells. MIF, median intensity fluorescence. All experiments were performed with at least 3 biological replicates. **p < 0.01; ***p < 0.001.

The enzymes responsible for glycosylation of extracellular proteins use activated monosaccharides, like UDP-GlcNAc and its derivates, UDP-GalNAc and CMP-Neu5Ac, as substrate. Thus, changes in the production of UDP-GlcNAc and its derivates could lead to changes in the glycan profile of the cells. To investigate this effect, we analyzed the expression of eight different saccharide epitopes in both cell lines (Figure 2A). When we compare each epitope between the two cell lines there are no significant changes in the expression of glycoconjugates for the majority of the epitopes analyzed, however two epitopes are significantly decreased in WM852 cells: the Tn antigen and the Sialyl Lewis a (SLe^A^) epitopes (Figure 2B).

### *O*-GlcNAcylation Is Reduced in WM852 Melanoma Cells

**Figure 3:**
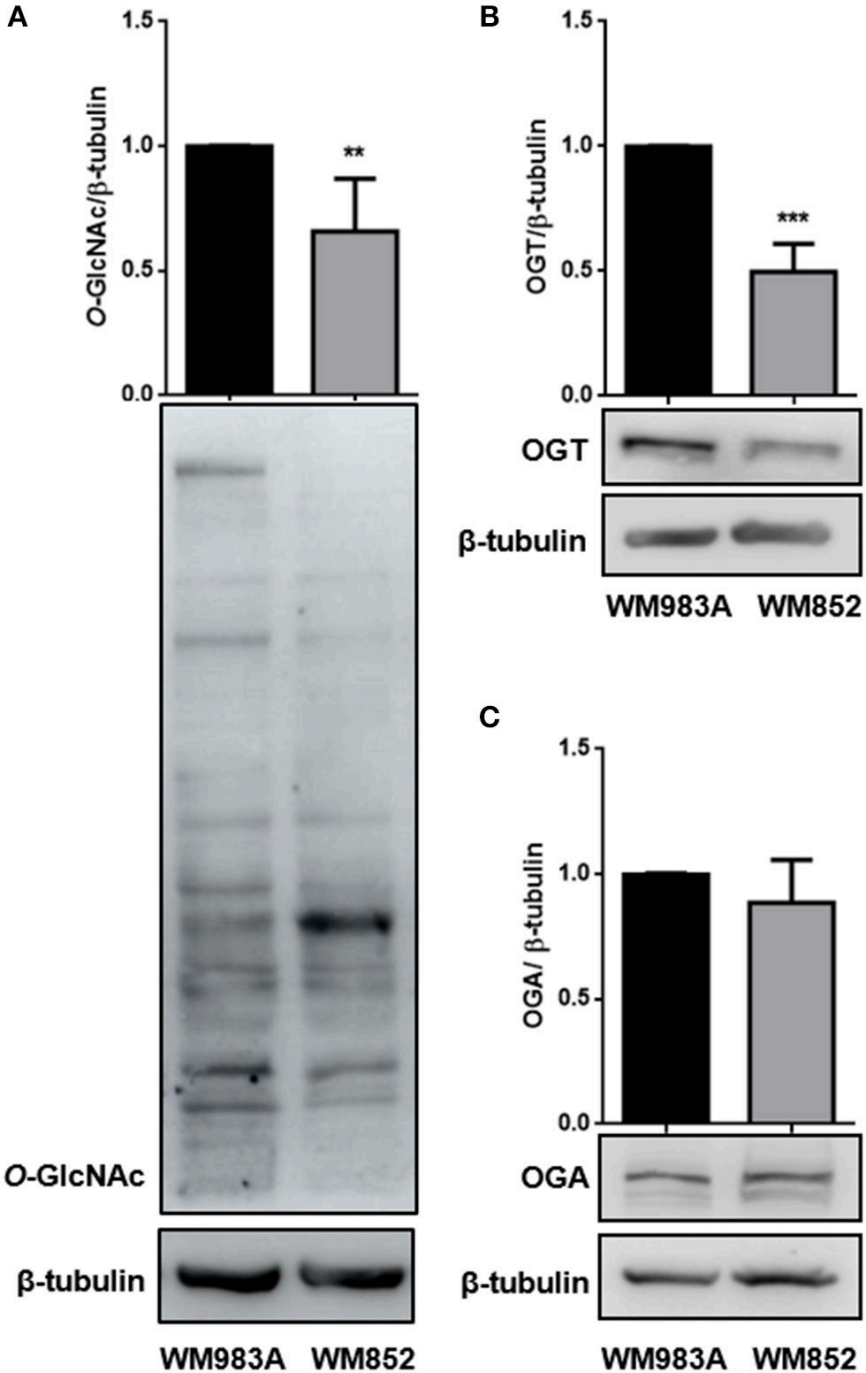
Characterization of *O*-GlcNAcylation in melanoma cell lines. Levels of **(A)** global *O*-GlcNAcylation, as well as **(B)** OGT and **(C)** OGA protein levels were measured by western blotting in WM983A (black bars) and WM852 (gray bars) cell lines. Quantification of protein levels in each cell line was normalized to β-tubulin. All experiments were performed with at least 3 biological replicates. **p < 0.01; ***p < 0.001.

The final product of HBP is used for O-GlcNAcylation. *O*-GlcNAcylation is characterized by the addition of and *N*-acetylglucosamine (GlcNAc) to serine and threonine residues. The enzyme *O*-GlcNAc transferase (OGT) adds a GlcNAc residue to proteins using UDP-GlcNAc as substrate, while the enzyme *O*-GlcNAcase (OGA) removes the modification (9). To investigate if lower HBP flux would lead to changes in the *O*-GlcNAc dynamic we analyzed the global *O*-GlcNAcylation in both cell lines and observed that WM852 cells present decreased protein *O*-GlcNAcylation compared to WM983A cells (Figure 3A). When the levels of the *O*-GlcNAc cycling enzymes (OGT and OGA), were analyzed we see reduced expression of OGT (Figure 3B) with no changes in the levels of OGA (Figure 3C), corroborating the decrease of proteins modified by *O*-GlcNAc in the WM852 cell line.

### Stimulation of HBP and *O*-GlcNAcylation Decreases Motility and Migration of Melanoma Cells

**Figure 4:**
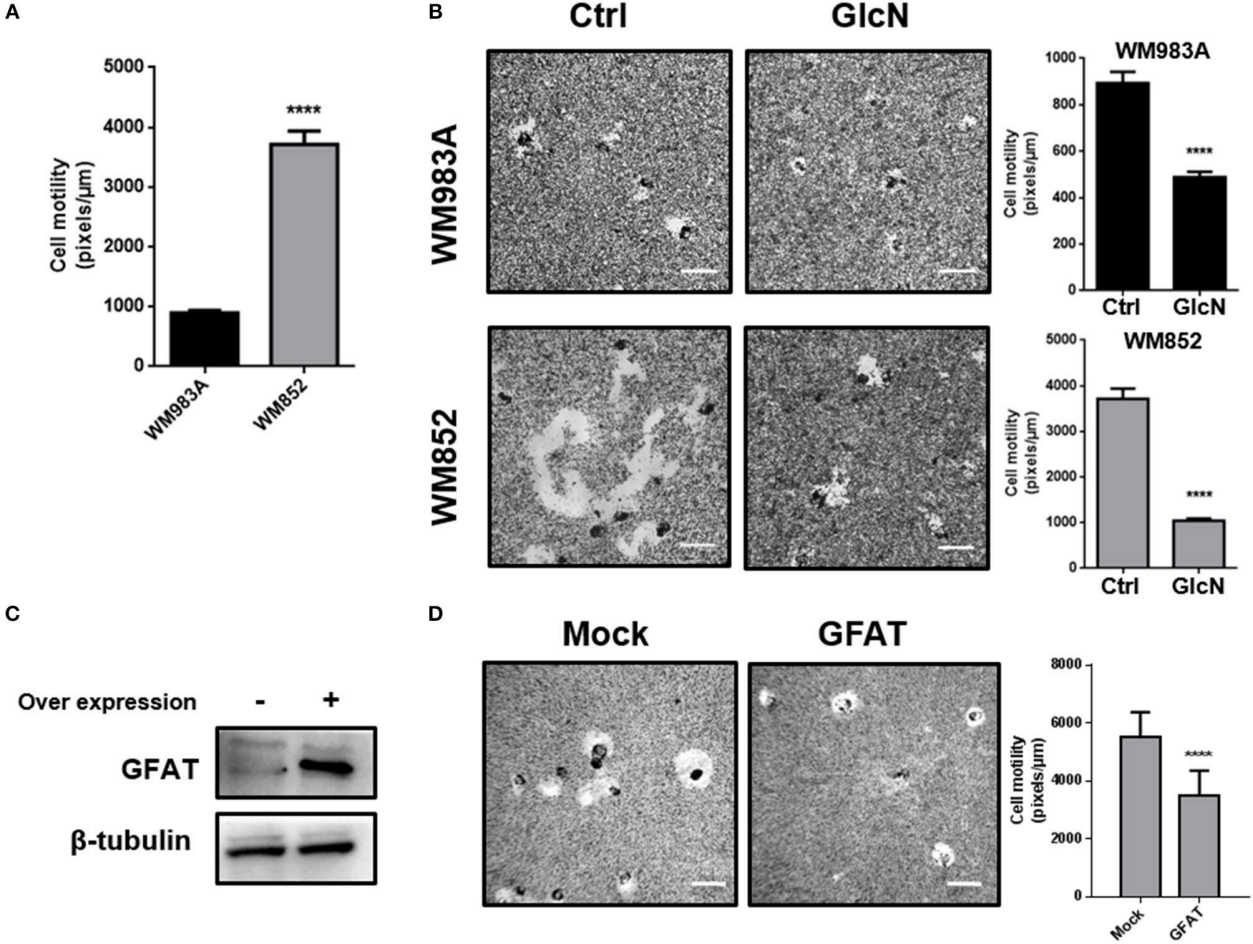
Increase in HBP's flux reduces cell motility in melanoma cells. **(A)** Comparison of cell motility between WM983A and WM852 melanoma cells. Bar graph quantifying single cell motility in WM983A (black bars) and WM852 (gray bars) cell lines. **(B)** Cell motility of melanoma cells in response to GlcN (10mM) treatment. Bar graph quantifying single cell motility in WM983A and WM852 cell lines and representative micrographs of motility track. **(C,D)** Cell motility of metastatic melanoma cells over expressing GFAT. **(C)** GFAT over expression in WM852 cells. **(D)** Bar graph quantifying single cell motility in WM852 cells and representative micrographs of motility track. All experiments were performed with at least 3 biological replicates. ****p < 0.0001.

In order to show the impact of HBP in melanoma cells, we use two strategies to increase HBP's flux: (1) treatment with glucosamine (GlcN), which bypass GFAT entering in the pathway as glucosamine-6-phosphate (the product of GFAT enzymatic activity), or (2) through over expression of GFAT. First, we measured cell motility through a single cell motility assay and we found that, as expected, WM852 cells possess higher motility than WM983A cells (Figure 4A). When HBP's flux is increased by treatment with GlcN we observe an important decrease in cell motility in both cell lines, but especially in WM852 (Figure 4B). To confirm this result, we over expressed GFAT in WM852 cells (Figure 4C). As observed in GlcN treated cells, GFAT over expression significantly decreases cell motility in WM852 cells (Figure 4D).

**Figure 5:**
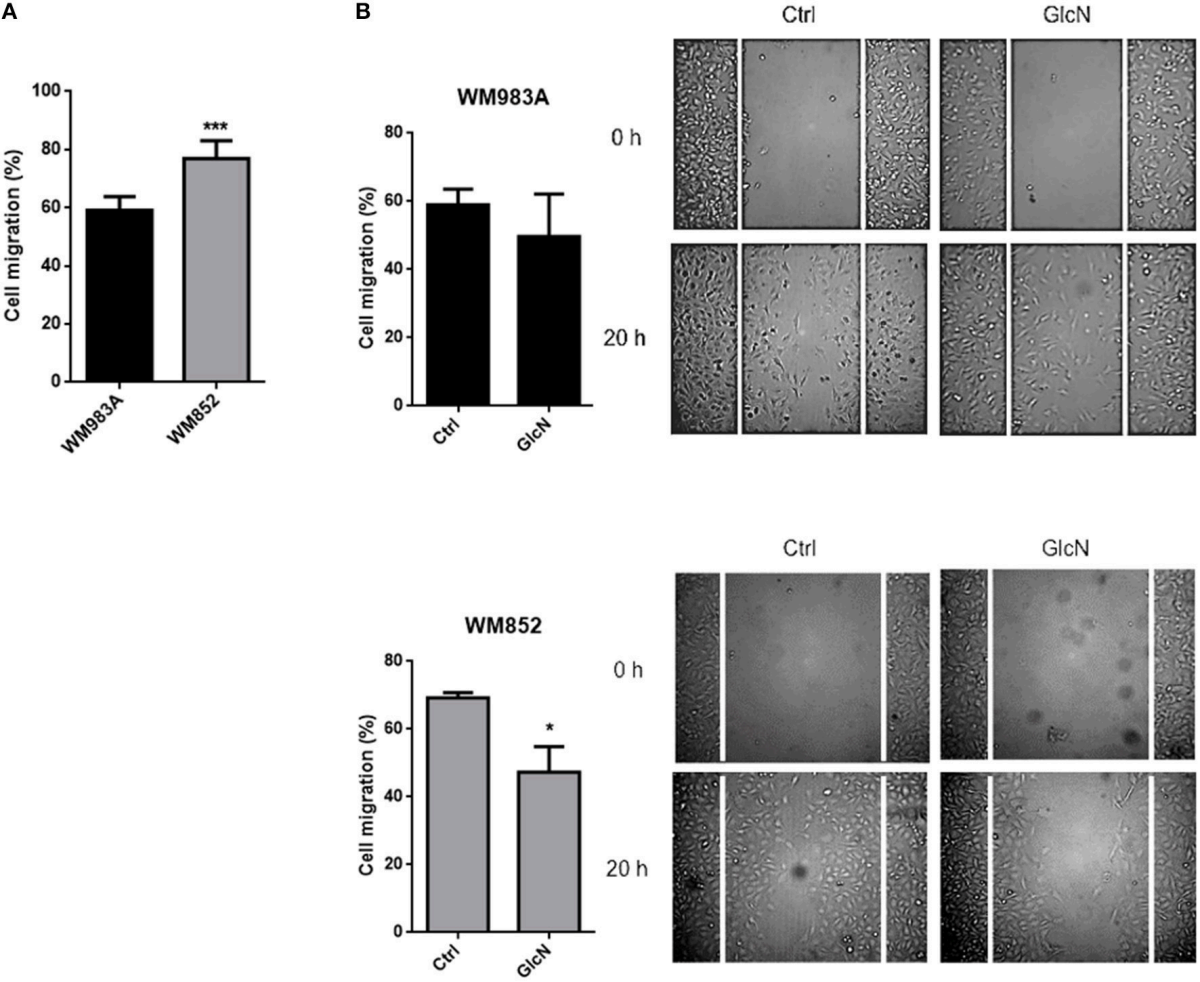
Increase in HBP's flux reduces cell migration in melanoma cells. **(A)** Comparison of cell migration in between WM983A and WM852 melanoma cells. Bar graph quantifying cell migration in WM983A (black bars) and WM852 (gray bars) cell lines and representative micrographs of wound closure. **(B)** Migration of melanoma cells in response to GlcN (10mM) treatment. Bar graph quantifying cell migration in WM983A and WM852 cell lines and representative micrographs of wound closure. Migration was measured by percentage of wound closure at final time (20 h) compared to time 0 (0 h), which was considered 0%. All experiments were performed with at least 3 biological replicates. *p < 0.05; ***p < 0.001.

We then analyzed cell migration in a monolayer where more aggressive cells have a higher rate of migration (Figure 5A). Treatment with GlcN decreases cell migration in both cell lines, especially in WM852 (Figure 5B). These data together show that HBP modulates cell motility and migration in melanoma cells regardless of HBP status and suggest that reduction of the flux at HBP may be a mechanism involved in the acquisition of a more aggressive phenotype in this model.

**Figure 6:**
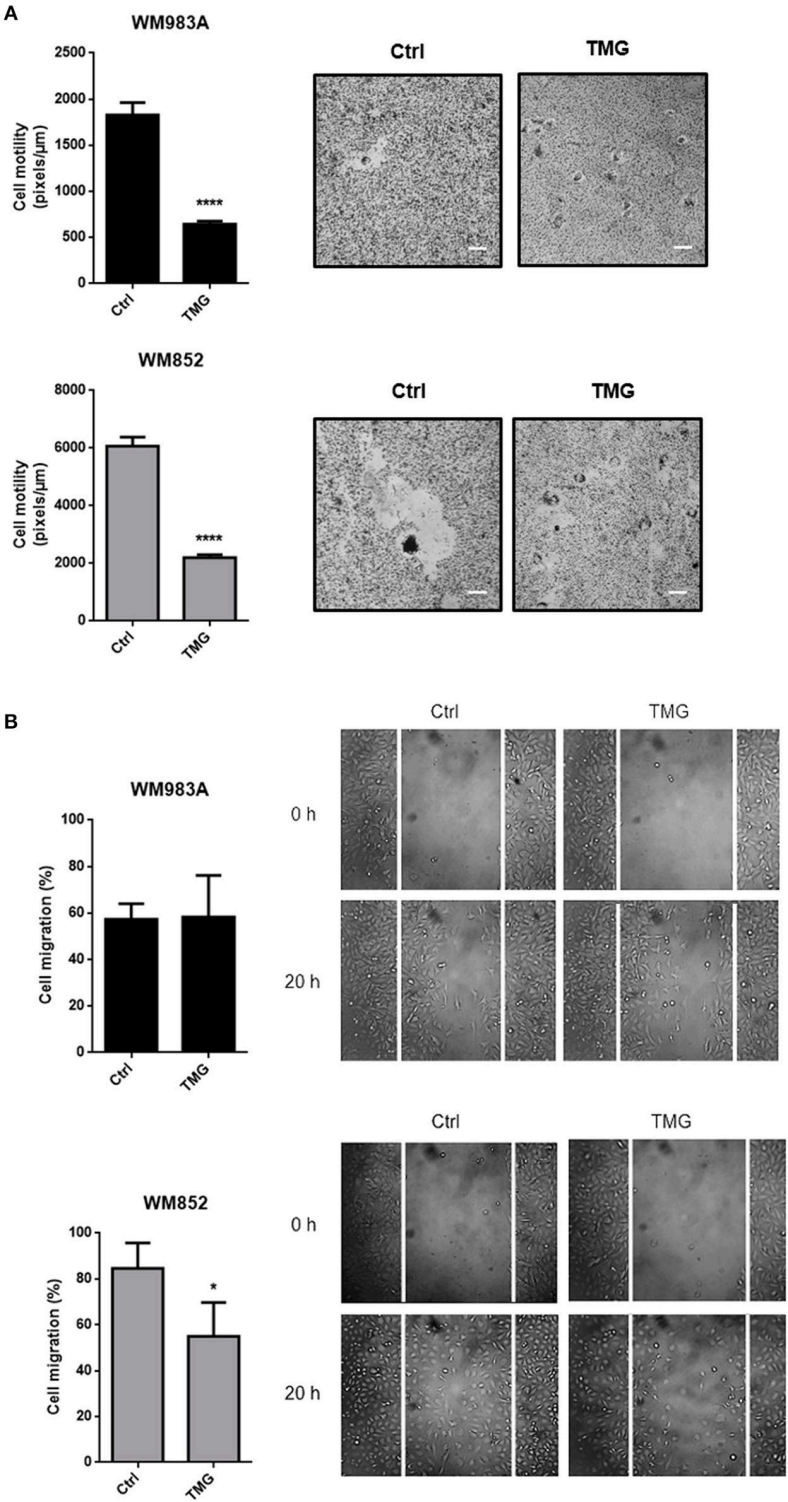
Hyper-*O*-GlcNAcylation reduces cell motility and migration in melanoma cells. **(A)** Cell motility of melanoma cells in response to TMG (10 μM) treatment. Bar graph quantifying single cell motility in WM983A and WM852 cell lines and representative micrographs of motility track. **(B)** Migration of melanoma cells in response to TMG (10 μM) treatment. Bar graph quantifying cell migration in WM983A and WM852 cell lines and representative micrographs of wound closure. Migration was measured by percentage of wound closure at final time (20 h) compared to time 0 (0 h), which was considered 0%. All experiments were performed with at least 3 biological replicates. *p < 0.05; ****p < 0.0001.

In order to test if the effects of up-regulation of HBP on cell motility and migration are due to *O*-GlcNAcylation, we induced hyper-*O*-GlcNAcylation using an OGA inhibitor Thiamet-G (TMG). The incubation of glucosamine and TMG is very well-established to increase the O-GlcNAcylation (Supplementary Figure 1). Similar to what happens when we increase HBP's flux, hyper-*O*-GlcNAcylation decreases single cell motility (Figure 6A) and cell migration (Figure 6B) in melanoma cells, especially in WM852 cells, suggesting that the mechanism by which HBP controls migration could involves the modulation of *O*-GlcNAcylation.

### *N*- and *O*-Glycosylation Are Important for Melanoma Cell Migration

**Figure 7:**
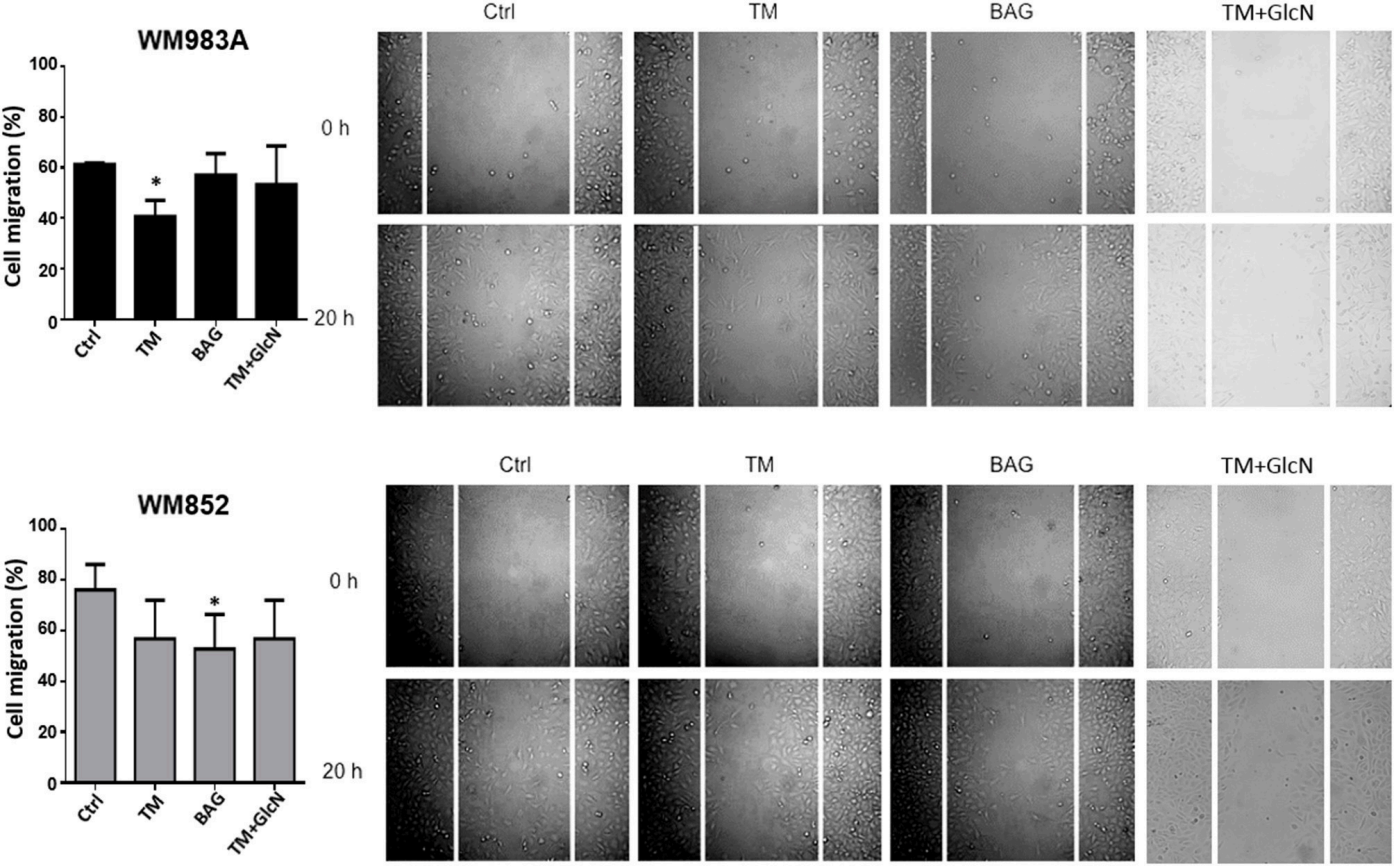
Inhibition of *N*- and *O*-GalNAc glycans reduces cell migration in melanoma cells. Migration of melanoma cells in response to Tunicamycin (TM) (1 μg/ml) and BAG (40 μM) and with GlcN (10 mM) and TM (1 μg/ml) treatments. Bar graph quantifying cell migration in WM983A (black bars) and WM852 (gray bars) cell lines and representative micrographs of wound closure. Migration was measured by percentage of wound closure at final time (20 h) compared to time 0 (0 h), which was considered 0%. All experiments were performed with at least 3 biological replicates. *p < 0.05.

Fluctuations of HBP's flux in general impact protein glycosylation. In Figure 2 we showed two downregulated epitopes in extracellular glycoconjugates from WM852 cells. To address the importance of extracellular glycosylation for cell migration we use pharmacological inhibitors of *N*-glycans and *O*-GalNAc glycans, Tunicamycin (TM) and benzyl2-acetamido-2-deoxy-α-D-galactopyranoside (BAG), respectively. Surprisingly, the inhibition of *N*-glycosylation was more efficient in reducing the migration of WM983A cells, while *O*-GalNAc glycans inhibition of WM852 cell line (Figure 7). In addition, we treat the cells with glucosamine and TM to interfere in HBP and *N*-glycosylation, respectively, and analyzed the cellular migration of melanoma cell types. Our results did not show a cumulative effect when interfering with both pathways. These data indicate that extracellular glycosylation is important for melanoma cell migration and demonstrate a different weight of importance of each type of extracellular glycosylation in each cell line.

### HBP and N-Glycosylation Regulates MMP Activity in Melanoma Cells

Degradation of the extracellular matrix (ECM) is an important step in cell invasion and metastasis, thus we decided to elucidate if HBP and the different types of glycosylation influence cell migration through modulation of matrix metalloproteases (MMPs) activity. MMPs are ECM-degrading proteases with different substrates along the components of the ECM. The activity of these enzymes is correlated to poor outcome in cancer (39–41). MMP-2 and -9 are secreted MMPs in the group of collagenases, also known as collagenases A and B, respectively, and appears to be associated with radial and vertical growth and with metastasis in melanoma (42).

**Figure 8:**
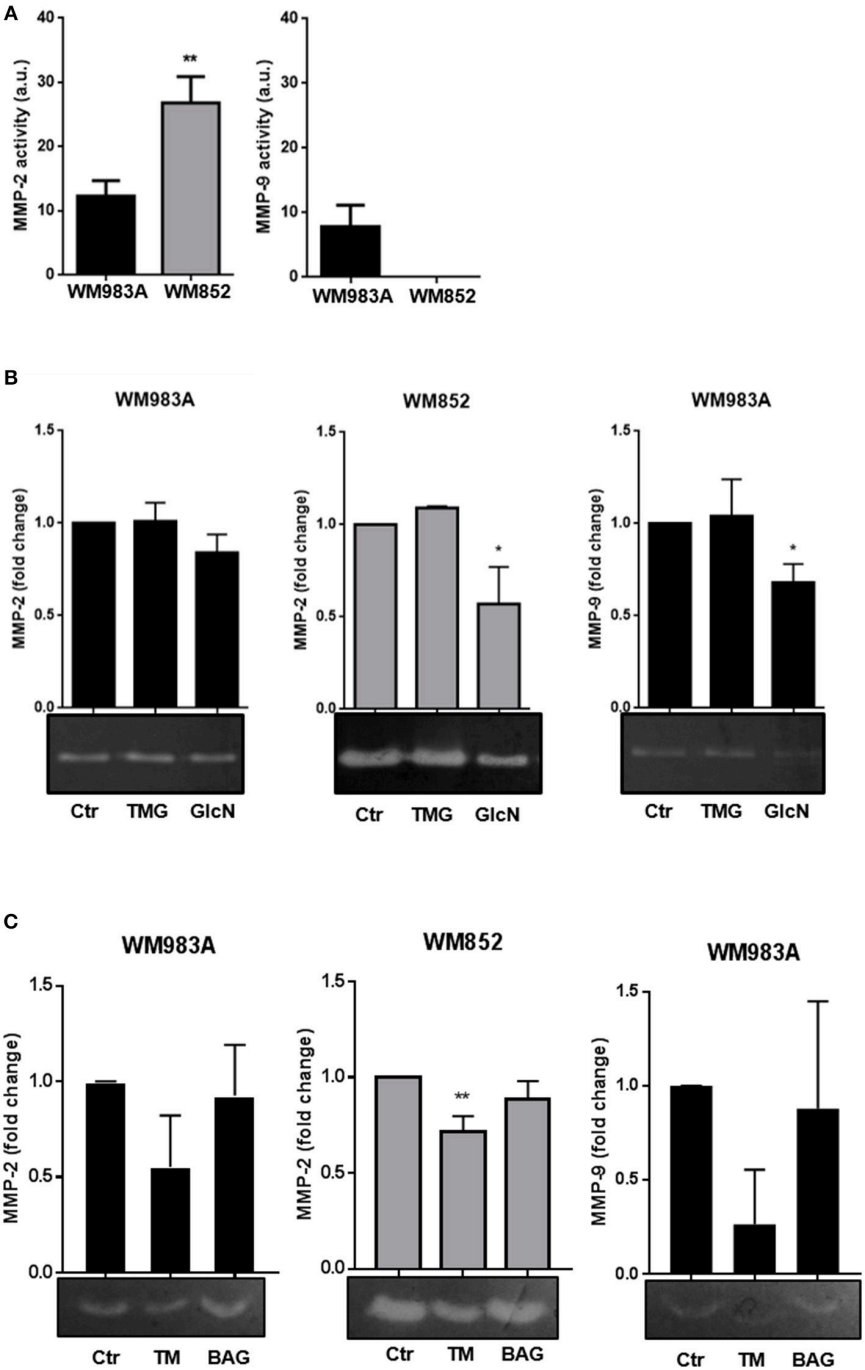
Increase in HBP's flux and N-glycosylation inhibition reduces MMP-2 and MMP-9 activities in melanoma cells. **(A)** Comparison of activity of MMP-2 and MMP-9 of WM983A and WM852 melanoma cells. Bar graphs quantifying MMP-2 and MMP-9 activities in WM983A (black bars) and WM852 (gray bars) cell lines. **(B)** Representative micrographs of zymography in response to GlcN (10 mM) and TMG (10 μM) treatments. Bar graph quantifying MMP-2 and MMP-9 of WM983A (black bars) and WM852 (gray bars) cell lines. **(C)** Activity of MMP-2 and MMP-9 in melanoma cells in response to Tunicamycin (TM) (1 μg/ml) and BAG (40 μM) treatments. Bar graph quantifying MMP-2 and MMP-9 of WM983A (black bars) and WM852 (gray bars) cell lines. Densitometry of zymography was normalized by the activity found in WM983A cells and represented as fold change. All experiments were performed with at least 3 biological replicates. *p < 0.05; **p < 0.01.

The activity of MMP-2 and MMP-9 in WM983A cells was described before (43), but the pattern of activity of these MMPs in WM852 cells is not known. We confirmed in WM983A cells activity of both MMP-2 and -9, with a higher activity of MMP-2 compared to MMP-9. Analysis of MMP-2 and MMP-9 activity in WM852 cells revealed a high activity of MMP-2 while the activity of MMP-9 was not detected in this cell line (Figures 8A,B). Comparing the two cell lines we observed higher activity of MMP-2 in the WM852 cell line (Figure 8A), as expected for metastatic cells compared to primary cells.

Regarding the influence of HBP in those activities, treatment with GlcN, which was shown to decrease cell migration, decreases MMP-2 and MMP-9 activities in both cell lines, especially in WM852 cells (Figure 8). However, treatment with TMG, which also decreases cell migration, had no effect on MMP activity (Figure 8B). The role of *N*-glycosylation and *O*-GalNAc glycans on MMPs activities was accessed by incubating the cells, respectively with TM and BAG (Figure 8C). Only *N*-glycosylation inhibition inhibits MMP-2 and MMP-9 activities from WM983A and WM852 cells. These data demonstrate that HBP regulates the activity of both collagenases in melanoma cells, pointing to a possible mechanism by which the pathway influences cell migration independently of *O*-GlcNAcylation and *O*-GalNAc glycans.

## Discussion

Metastasis is the main cause of death among oncologic patients worldwide, thus understanding the mechanisms involved in this process can provide insights into future targets for new therapies. Especially in advanced stage melanoma, the traditional chemo- and radiotherapies strategies generally used to treat patients with metastasis are ineffective. HBP and *O*-GlcNAcylation involvement in tumor-related processes have been studied recently and linked to tumor cell proliferation and metastasis in different tumor types (26, 33, 44), but very few is known in melanoma. In this work we focus on the analysis of HBP and the three major types of glycosylation (*O*-GlcNAcylation, *N*- glycosylation and *O*-GalNAc glycans) in human cell lines derived from patients with melanoma and its influence in cell migration and MMPs, important characteristics for metastasis development. We showed that an increase in HBP's flux, as well as increased *O*-GlcNAcylation, decreases cell motility of both melanoma cells. In addition, inhibition of extracellular glycosylation leads to the same phenotype. Finally, we observed that modulation of HBP, but not *O*-GlcNAcylation, impacts MMP's activity suggesting different mechanisms of regulation.

We analyzed the status of HBP by GFAT expression, since it is poorly known if GFAT1 or GFAT2 are expressed in skin cells. GFAT1 is described as widely expressed so it was expected that skin cells express the enzyme, while GFAT2 is significantly expressed only in specific cell types, notably the reproductive system, adipose tissue, smooth muscle, nervous system and spinal cord (38, 45). RNA-seq analysis of GFAT1 and GFAT2 gene expression (encoded by the genes *GFPT1* and *GFPT2*, respectively) in patient samples show significant expression of GFAT1 in skin and low or no expression of GFAT2 (45–47). In accordance, a cell line of human keratinocytes (HaCaT) presents a significant amount of mRNA for GFAT1 but undetectable levels of mRNA for GFAT2 (48). However, transformed skin fibroblasts present increased expression of GFAT2, but no alterations in GFAT1 expression compared to normal skin tissue (45). Regarding HBP, most tumor types in which this pathway was analyzed present increased expression of HBP's rate-limiting enzyme, GFAT (6). However, in other tumors like gastric cancer the opposite happens, where a decrease in GFAT expression is found in tumors compared to normal tissue, and the expression of the enzyme is associated with a good prognosis of the disease (30). Here, we showed that both expression level and activity of GFAT1 is decreased in the metastatic cell line in comparison with the primary line. The same was observed recently by Deen and colleagues when analyzing normal melanocytes and other two melanoma cell lines as well as biopsies samples taken from normal nevi and melanoma patients. They show that GFAT1 expression decreases as the aggressiveness increase from primary melanocytes to more metastatic melanoma cells (49). Additionally, our work demonstrates for the first time that GFAT2 have the same trend of decrease, suggesting that regulation of HBP pathway contributes to the acquisition of the metastatic phenotype. Indeed, we used two approaches, GFAT over-expression or glucosamine incubation to show that an increase of HBP flux cause the reduction of motility/migration in both melanoma cell lines.

Discrepancies also happen when *O*-GlcNAcylation is analyzed among tumor types, where the majority of tumor types present hyper-*O*-GlcNAcylation (9), while others show decreased *O*-GlcNAcylation in tumors compared to normal tissue (50–52). We showed that *O*-GlcNAcylation is also decreased in WM852 cells, as well as OGT expression, with no changes in OGA levels. Jiang and colleagues using patient samples of invasive ductal breast carcinoma describe the same pattern: a decrease in global protein *O*-GlcNAcylation and OGT expression with no changes in OGA levels in samples from patients that evolved to lymph node metastasis in comparison to non-metastatic patients (53). In addition, patients with a higher number of affected lymph nodes presented lower *O*-GlcNAcylation and lower OGT expression but no difference in OGA levels when compared to metastatic lymph nodes from patients with a lower number of metastatic foci (53). *O*-GlcNAc is shown to modulate expression of MMP-2 and -9 in some cells, but not in others (54, 55). In our model, while modulation of HBP decreases activity of MMPs, modulation of *O*-GlcNAc does not suggesting that other kind of glycosylation should modulate MMPs.

Thus, we investigated the impact of *N*-glycosylation and *O*-GalNAc glycans in the regulation of MMP activity. The importance of *N*-glycosylation for metastasis in melanoma is described in the literature (56–59). Interestingly, while inhibition of *N*-glycosylation is more efficient in reducing the migration of WM983A cells, O-GalNAc glycans inhibition compromised the migration of WM852 cell line. More experiments are needed to determine if those differences are due to the disease stage (primary tumor/metastasis) or due to cell line-specific differences. Curiously, the only two epitopes differentially expressed in WB852 cells are Tn antigen and Sialyl Lewis^A^ (SLe^A^). To our knowledge, this is the first time that a high expression of Tn antigen is reported in melanoma cells. Although Tn antigen is differently expressed by WM983A and WM852 cells, this epitope is important for some cells during the metastatic process but not required for others (51). Because tumor proteins bearing Tn carbohydrates represent a potential target for immunotherapy (52), our observation lays the foundation to study Tn antigen as a target for melanoma immunotherapy or vaccination. SLeA is responsible for the E-selectin-mediated adhesion of human cancer cells to the endothelium, and it is present in high levels on the surface of human pancreatic, colon and gastric cancer cell lines (17). On the other side, normal epithelial cells express low amounts of SLeA antigen (53, 54). Recently we demonstrated, in mouse colon adenocarcinoma MC38 cells, that GFAT downregulation significantly decreased the expression of Tn antigens (VVL binding) and MAA binding (17). The decreased expression of Tn and SLeA epitopes in WM852 cell, in which the expression and activity of GFAT1 and 2 are significantly decreased is consistent with the participation of HBP in aberrant glycosylation in melanoma cells.

In conclusion, in this work we characterized and showed the importance of HBP and the three major types of glycosylation for cell motility/migration and MMPs, using two melanoma cell lines. The data from this work together with what is found in the literature reinforce the idea that HBP and glycosylation is an important process in the tumor cell machinery and that different types of glycosylation can have different roles in cells, thus they might be considered as independent pathways. Together, our results indicate that HBP and glycosylation emerge as a possible target for therapeutic strategies in clinic.

## Author Contributions

RdQ, MC, AT, BD, and WD conceived the idea for the project. RdQ, IO, BP, FB, BdC, and AdC conducted the experiments. RdQ, MC, AT, and WD analyzed the results. RdQ wrote the paper. IO, BP, MC, AT, BD, and WD contributed to manuscript preparation. All authors reviewed the results and approved the final version of the manuscript.

### Conflict of Interest Statement

The authors declare that the research was conducted in the absence of any commercial or financial relationships that could be construed as a potential conflict of interest.
