# Supplementary material for: Hexosamine Biosynthetic Pathway and Glycosylation Regulate Cell Migration in Melanoma Cells
Source: Front Oncol. 2019 Mar 5;9:116. doi: 10.3389/fonc.2019.00116 (PMC6411693; doi:10.3389/fonc.2019.00116)
Supplement: Supplementary file 1 [file Presentation_1.PPTX]

## Slide 1
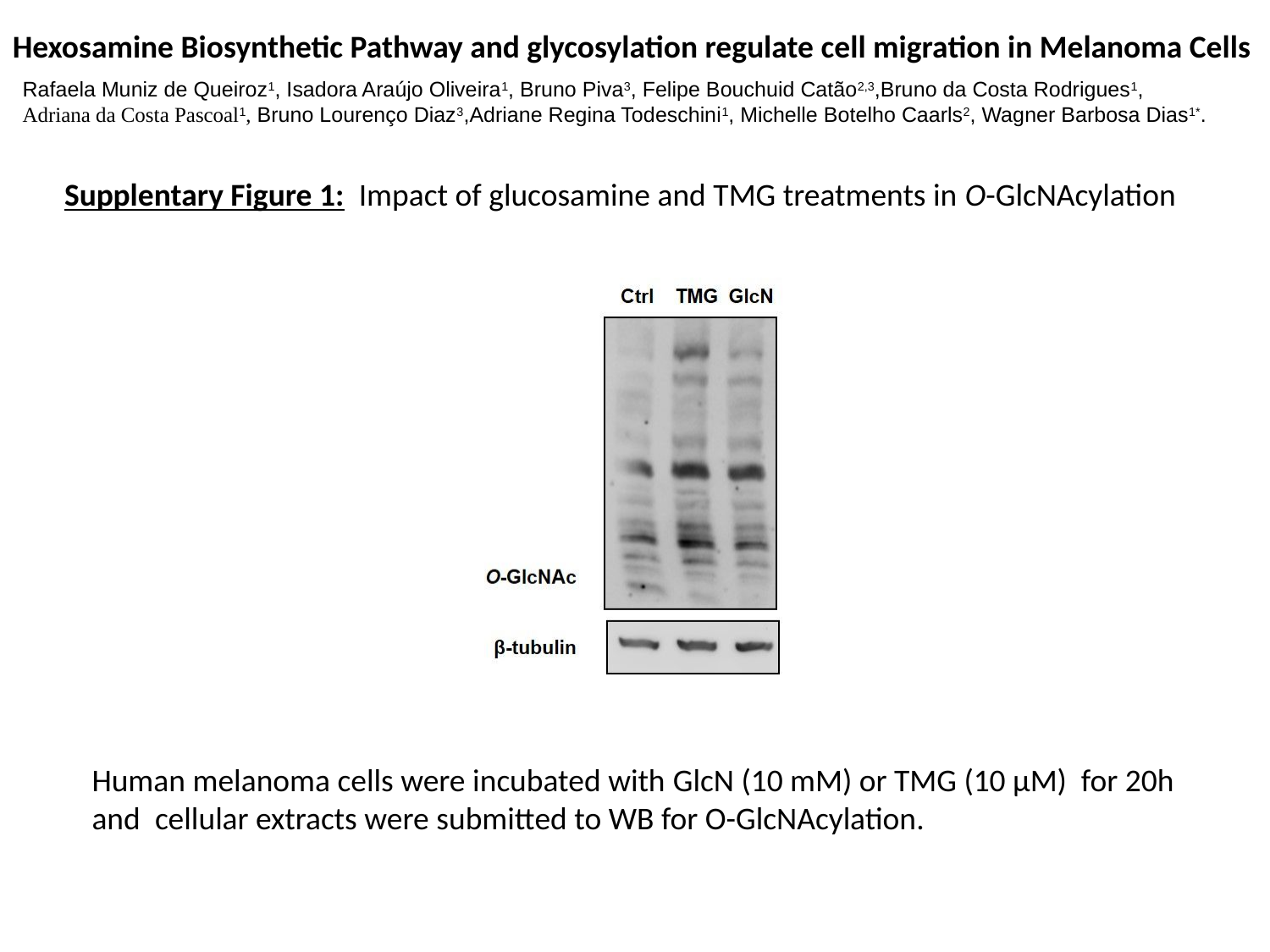

Hexosamine Biosynthetic Pathway and glycosylation regulate cell migration in Melanoma Cells
Rafaela Muniz de Queiroz1, Isadora Araújo Oliveira1, Bruno Piva3, Felipe Bouchuid Catão2,3,Bruno da Costa Rodrigues1, Adriana da Costa Pascoal1, Bruno Lourenço Diaz3,Adriane Regina Todeschini1, Michelle Botelho Caarls2, Wagner Barbosa Dias1*.
Supplentary Figure 1: Impact of glucosamine and TMG treatments in O-GlcNAcylation
Human melanoma cells were incubated with GlcN (10 mM) or TMG (10 µM) for 20h and cellular extracts were submitted to WB for O-GlcNAcylation.
